# Dishevelled-Associated Activator of Morphogenesis 2 (DAAM2) Predicts the Immuno-Hot Phenotype in Pancreatic Adenocarcinoma

**DOI:** 10.3389/fmolb.2022.750083

**Published:** 2022-02-24

**Authors:** Qinglin Zhang, Jiadong Pan, He Nie, Hui Wang, Fangmei An, Qiang Zhan

**Affiliations:** Department of Gastroenterology, Wuxi People’s Hospital Affiliated to Nanjing Medical University, Wuxi, China

**Keywords:** DAAM2, tumor immunity, pancreatic adenocarcinoma, bioinformatics, biomarker

## Abstract

**Background:** DAAM2 participates in the oncogenesis and progression of human cancers. Although the role of DAAM2 in cancers has been preliminarily investigated, its correlations with antitumor immunity are unclear.

**Methods:** A pancancer analysis was conducted to explore the immunological role of DAAM2 based on RNA sequencing (RNA-seq) data downloaded from The Cancer Genome Atlas (TCGA). Next, correlations between DAAM2 and immunological characteristics in the tumor microenvironment (TME) of pancreatic adenocarcinoma (PAAD) were evaluated. In addition, the role of DAAM2 in predicting the clinical characteristics and the response to various therapies in PAAD were also assessed. In addition, the correlations between DAAM2 and the emerging immunobiomarker N6-methyladenosine (m6A) genes were also evaluated.

**Results:** Pancancer analysis revealed that DAAM2 exhibited positive correlations with a majority of immunomodulators, tumor-infiltrating immune cells (TIICs) and inhibitory immune checkpoints in several cancer types, including PAAD. In addition, DAAM2 was associated with an inflamed phenotype in the tumor microenvironment (TME). DAAM2 also predicted significantly higher responses to chemotherapy, anti-EGFR therapy and immunotherapy but lower responses to anti-ERBB2 and antiangiogenic therapy. In addition, DAAM2 was correlated with immune-related microbiota.

**Conclusion:** In PAAD**,** DAAM2 is associated with an immuno-hot phenotype and can help predict the outcome of various therapeutic options. Overall, DAAM2 is a promising indicator for assessing high immunogenicity in PAAD.

## Background

Pancreatic adenocarcinoma (PAAD) is a common malignancy featuring deadly aggressiveness and high mortality (Rawla et al., 2019). The 5-years survival rate of PAAD patients receiving surgical treatment is approximately 10–25% (Jiang et al., 2021). Unfortunately, more than 80% of patients with PAAD are diagnosed with unresectable status in the first evaluation (Okasha et al., 2017), and these patients face a worse prognosis. Recently, immunotherapy has emerged as a promising therapeutic strategy for PAAD (Banerjee et al., 2018). In principle, the response to immunotherapy largely depends on the tumor microenvironment (TME), which consists of immune cells, stromal cells, vascular networks, and many other cellular and noncellular components (Duan et al., 2020). According to the characteristics of the TME, tumors can be divided into hot and cold tumors. Immuno-hot tumors are characterized by T cell infiltration and molecular signatures of immune activation and exhibit a higher response to various therapies, including immunotherapy (Duan et al., 2020). Therefore, potential biomarkers that could be used to identify tumor immunogenicity are significant for the demarcation of populations with advantages for immunotherapy.

The dishevelled-associated activator of morphogenesis (DAAM) gene family is a subfamily of Formin proteins (Matusek et al., 2008; Prokop et al., 2011) consisting of DAAM1 and DAAM2 (Ajima et al., 2015). DAAM1 is essential for cancer progression. DAAM1 was overexpressed in breast cancer and promoted cancer metastasis in response to Wnt5a (Zhu et al., 2012; Mei et al., 2019; Mei et al., 2020b). Although DAAM2 is rarely studied in cancer, its significant role in tumor progression is nonnegligible. Zhu et al. (2017) reported that DAAM2 accelerated glioma formation by facilitating ubiquitination and degradation of Von Hippel-Landau (VHL) protein. In addition, DAAM2 promoted the progression of hepatocellular carcinoma by enhancing hypoxia-inducible factor 1α (HIF-1α) expression (Fang et al., 2020). It has been widely proven that hypoxia is tightly associated with antitumor immunity in most cancer types (Abou Khouzam et al., 2020; Fu et al., 2021). However, the potential correlation between DAAM2 and immunological features in human cancers has not yet been explored.

In the current study, the expression and immunological role of DAAM2 across cancers were first analyzed. The findings revealed that DAAM2 showed tight correlations with immunological factors in most cancers, but the highest correlation was found with PAAD. In addition, DAAM2 identified an inflamed TME and increased TIIC abundance in PAAD, namely, immuno-hot tumors. In addition, DAAM2 has the potential to predict therapeutic efficacy in various therapies. Overall, DAAM2 is a promising biomarker for identifying high immunogenicity in PAAD.

## Materials and Methods

### Public Data Acquisition

The pancancer normalized RNA sequencing (RNA-seq) data and clinical annotations were obtained from UCSC Xena (https://xenabrowser.net/datapages/). The abbreviations for various cancer types are given in Supplementary Table S1. To explore microorganisms associated with antitumor immunity in PAAD, we downloaded relative abundance information of ∼1,400 microorganisms from the online data portal ciboPortal (http://www.cbioportal.org/) (Cerami et al., 2012) and selected the microorganisms with absolute Pearson’s correlations with the T cell inflamed score were ≥0.3 or ≤ −0.3 and with *p* values ≤0.05. In addition, Buffa, Winter and Ragnum hypoxia scores for PAAD samples were also downloaded from the ciboPortal.

### Prediction of DAAM2 Classifier Genes and Clustering Analysis in Pancreatic Adenocarcinoma

To identify DAAM2 classifier genes that are positively or negatively coexpressed with DAAM2 in the normalized RNA-seq data of PAAD from the TCGA database, we used the biweight midcorrelation (bicor) algorithm to evaluate “similarity” between gene expression profiles, which is thought to be a good measurement for gene coexpression module analysis (Zheng et al., 2014). First, the bicor algorithm was used to measure the “similarity” between DAAM2 and genes with mean expression values >1, and the 99th and first percentiles of the bicor values were defined as the cutoffs of positive and negative coexpression with DAAM2, which were 0.6921602 and −0.4860878, respectively. Then, 176 genes positively correlated with DAAM2 and 176 genes negatively correlated with DAAM2 were identified. After identifying DAAM2 classifier genes, nonnegative matrix factorization (NMF), an unsupervised dimension reduction technique, was utilized to classify PAAD patients in each cohort into two subtypes (High-DAAM2 and Low-DAAM2) based on the nonsmooth NMF algorithm from Pascual-Montano (Mejia-Roa et al., 2015).

### Gene Function Annotation and Pathway Enrichment Analysis

DAVID (https://david.ncifcrf.gov/) is a widely used gene functional annotation website (Dennis et al., 2003). In this study, DAVID was applied to perform Gene Ontology (GO) and Kyoto Encyclopedia of Genes and Genomes (KEGG) analyses of 176 genes positively correlated with DAAM2 and 176 genes negatively correlated with DAAM2. The human genome (Homo sapiens) was selected as the background variable. Enrichment terms were considered statistically significant when the *p* values were less than 0.05, and the top 10 terms of each analysis were retained.

### Evaluation of the Immunological Characteristics of the Tumor Microenvironment

Given that immune and tumor cells were both present in the tissues that were subjected to RNA-seq, we next explored the differences in various immunological characteristics between High-DAAM2 and Low-DAAM2 subtypes referring to previous research (Cai et al., 2021).

Information on 122 immunomodulators, well-known effector genes of tumor-infiltrating immune cells (TIICs), and 18 specific genes correlated with T cell inflammation and their weighting coefficients was collected from previous studies (Ayers et al., 2017; Charoentong et al., 2017). In addition, the assessment index of tissue from each patient, including the tumor purity, ESTIMATE score, immune score and stromal score of each patient, was estimated using the ESTIMATE algorithm (Yoshihara et al., 2013). Moreover, to avoid the miscalculation caused by various algorithms when estimating the levels of TIICs, we comprehensively computed the relative abundance of TIICs using the following independent algorithms: TIMER (Li et al., 2020), EPIC (Racle et al., 2017), MCP-counter (Becht et al., 2016), quanTIseq (Finotello et al., 2019) and TISIDB (Ru et al., 2019). Considering that each stage of the cancer-immune cycle plays a crucial role in reflecting the anticancer immune response and determining the destiny of tumor cells, we next calculated the activities of each stage by single-sample gene set enrichment analysis (ssGSEA) according to the expression level of stage-specific signatures (Xu et al., 2018).

### Prediction of Therapeutic Response

The role of DAAM2 in predicting the response to chemotherapy was also estimated. We extracted PAAD-related drug targets by searching the DrugBank database and compared the difference in their expression between the high- and low-DAAM2 groups.

### Calculation of the Enrichment Scores of Various Gene Signatures

Referring to previous research (Hu et al., 2021), we collected several gene sets positively correlated with antitumor immunity, such as genes involved in DNA replication and hypoxia, and gene sets that predicted therapeutic response, such as EGFR ligands. The enrichment scores of these signatures were calculated by the ssGSEA algorithm (Hanzelmann et al., 2013).

### Clinical Samples

Two PAAD tissue microarrays (TMAs, HPanA150CS04 and HPanA150CS02) were obtained from Outdo Biotech (Shanghai, China). The HPanA150CS04 microarray contained 120 PAAD and 30 paratumor samples. The HPanA150CS02 microarray contained 78 PAAD and 72 paratumor samples. Ethical approval for the study of TMA was granted by the Clinical Research Ethics Committee, Outdo Biotech (Shanghai, China).

### Immunohistochemistry and Semiquantitative Scoring

Immunohistochemistry (IHC) staining was directly conducted on the HPanA150CS04 TMA using standard procedures. A primary antibody for DAAM2 (1:200 dilution, Cat. 25206-1-AP, ProteinTech, Wuhan, China) was used to detect DAAM2 expression. Antibody staining was visualized with DAB and hematoxylin counterstain, and stained sections were scanned using Aperio Digital Pathology Slide Scanners. The stained TMA was independently assessed by two pathologists. For semiquantitative assessment of DAAM2 staining, the immunoreactivity score (IRS) was used according to a previous description (Mei et al., 2019; Mei et al., 2020b).

### Cell Culture and Transfection

CFPAC-1 (Cat. KG176) cells were purchased from KeyGEN (Nanjing, China) and cultured at 37°C with 5% CO_2_. CFPAC1 cells were cultured in IMDM supplemented with 10% fetal bovine serum (FBS). All experiments were performed with mycoplasma-free cells. In addition, CFPAC-1 cells have recently been authenticated using short tandem repeat profiling.

For DAAM2 knockdown, CFPAC-1 cells were cultured to ∼70% confluence in 6-well plates and transfected with siRNA-NC (5′-UUC​UCC​GAA​CGU​GUC​ACG​UTT-3′) or siRNA-DAAM2 (5′-GAC​CGC​UUC​CUC​UAU​GAA​ATT-3′) using Lipofectamine 3000 Reagent (Cat. L3000015, Invitrogen, Carlsbad, CA, United States). The transfection efficiency was validated using Western blotting analysis.

### Western Blotting Analysis

Total proteins of cells were harvested using lysis buffer. Then, SDS-polyacrylamide gel electrophoresis (SDS–PAGE) and Western blotting analysis were conducted according to standardized protocols. The primary antibodies used were as follows: DAAM2 (1:500 dilution, Cat. 25206-1-AP, ProteinTech), PD-L1 (1:1000 dilution, Cat. 66248-1-Ig, ProteinTech) and β-actin (1:2000 dilution, Cat. 66009-1-Ig, ProteinTech). The protein level of DAAM2 was standardized to that of β-actin.

### Statistical Analysis

Pearson’s correlation coefficient was used for correlation analysis. The chi-square exact test was utilized for categorical variables, while the Wilcoxon rank-sum test was performed to measure the differences in continuous variables between groups. All tests were two-sided and were conducted in R version 3.6.0, and a *p* value ≤0.05 was considered statistically significant if not noted. Statistical significance was defined as *p value ≤0.05, **p value ≤0.01, ***p value ≤0.001, and ****p value ≤0.0001.

## Results

### Analysis of the Immunological Correlation of DAAM2 Across Cancers

**FIGURE 1:**
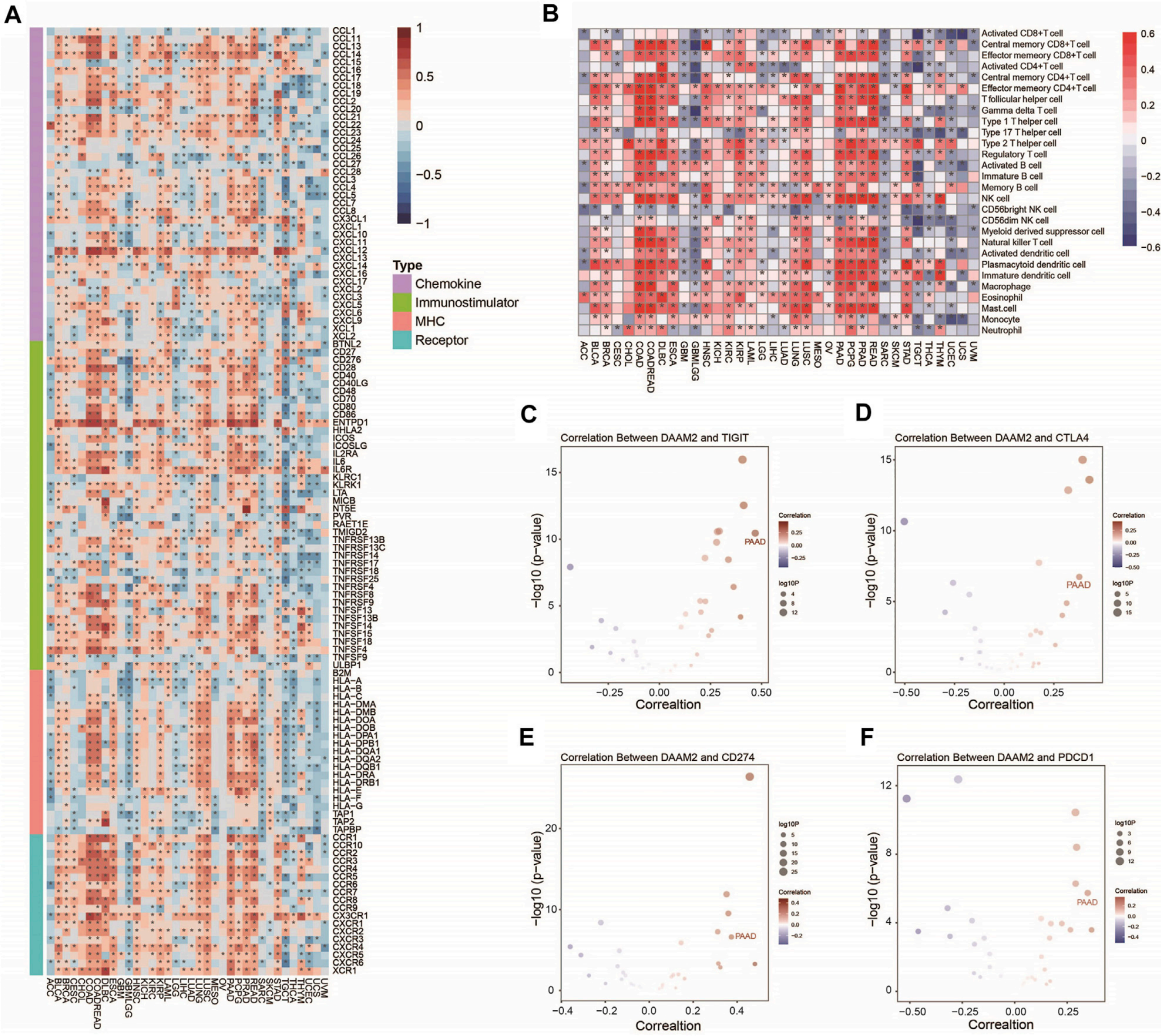
Pancancer analysis of the effect of DAAM2 on immunological status. **(A)** Correlations between DAAM2 and 122 immunomodulators. The color indicates the correlation coefficient. The asterisks indicate significant differences assessed by Pearson analysis. **(B)** Correlations between DAAM2 and 28 TIICs calculated with the ssGSEA algorithm. The color indicates the correlation coefficient. The asterisks indicate significant differences assessed by Pearson analysis. **(C–F)** Correlation between DAAM2 and the immune checkpoints TIGIT, CTLA4, CD274 and PDCD1. The dots represent cancer types. The *Y*-axis represents the Pearson correlation coefficient, while the *X*-axis represents -log_10_ (*p* value).

We first performed a pancancer analysis to investigate the immunological role of DAAM2 in all accessible tumor types in the TCGA database. The results suggested that DAAM2 exhibited positive correlations with a majority of immunomodulators in several cancer types, particularly in gastrointestinal tumors (Figure 1A). We next calculated the infiltrating levels of TIICs in the TME using the ssGSEA method. Similarly, DAAM2 expression was highly correlated with most types of TIICs in most gastrointestinal tumors, such as COAD, ESCA, PAAD and READ (Figure 1B). We also assessed the correlations between DAAM2 and the expression of immune checkpoints, including TIGIT, CTLA4, CD274, and PDCD1, across cancers. The results showed that DAAM2 was positively correlated with these immune checkpoints in multiple cancers, and a higher correlation was observed in PAAD (Figures 1C–F). Collectively, these results uncover the potential role of DAAM2 as an immune-related indicator in human cancers, especially PAAD.

### Expression of DAAM2 in Pancreatic Adenocarcinoma Tissues

**FIGURE 2:**
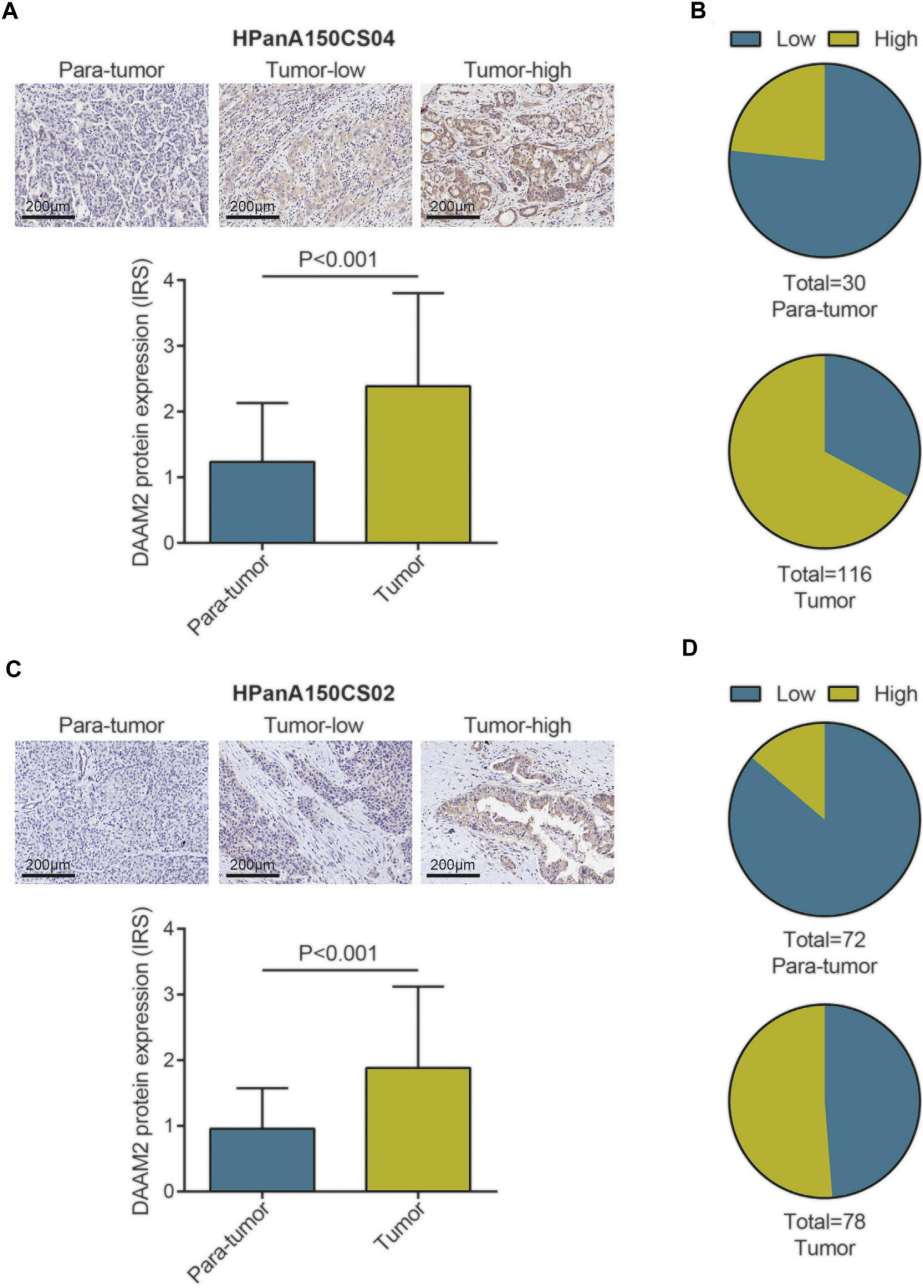
DAAM2 expression in PAAD tissues and normal pancreatic tissues. **(A)** Representative microphotographs represent low and high staining intensity in PAAD tissues and normal tissues (HPanA150CS04). Brown, DAAM2. Blue, hematoxylin. **(B)** DAAM2 protein expression intensity proportion of PAAD tissues and paired normal tissues (HPanA150CS04). Low expression: IRS < 2; High expression: IRS ≥ 2. **(C)** Representative microphotographs represent low and high staining intensity in PAAD tissues and normal tissues (HPanA150CS02). Brown, DAAM2. Blue, hematoxylin. **(D)** DAAM2 protein expression intensity proportion of PAAD tissues and paired normal tissues (HPanA150CS02). Low expression: IRS < 2; High expression: IRS ≥ 2.

We subsequently compared the expression of DAAM2 in PAAD and paratumor tissues. Two TMAs of PAAD tissues were submitted for IHC staining. As shown in Figure 2A, the immunoreactivity of DAAM2 was mostly localized to the cytoplasm. After analysis of the HPanA150CS04 TMA, the IRS of DAAM2 in PAAD tissues was significantly enhanced compared with that in paired paracancerous tissues (Figure 2A). In addition, we found that the samples with high DAAM2 expression accounted for a majority of tumor tissues (Figure 2B). Moreover, the analysis of HPanA150CS02 TMA was also in accord with the above results (Figures 2C,D). Overall, these results revealed that DAAM2 was upregulated in PAAD tissues.

### Subgrouping Pancreatic Adenocarcinoma Patients Based on DAAM2 Expression

**FIGURE 3:**
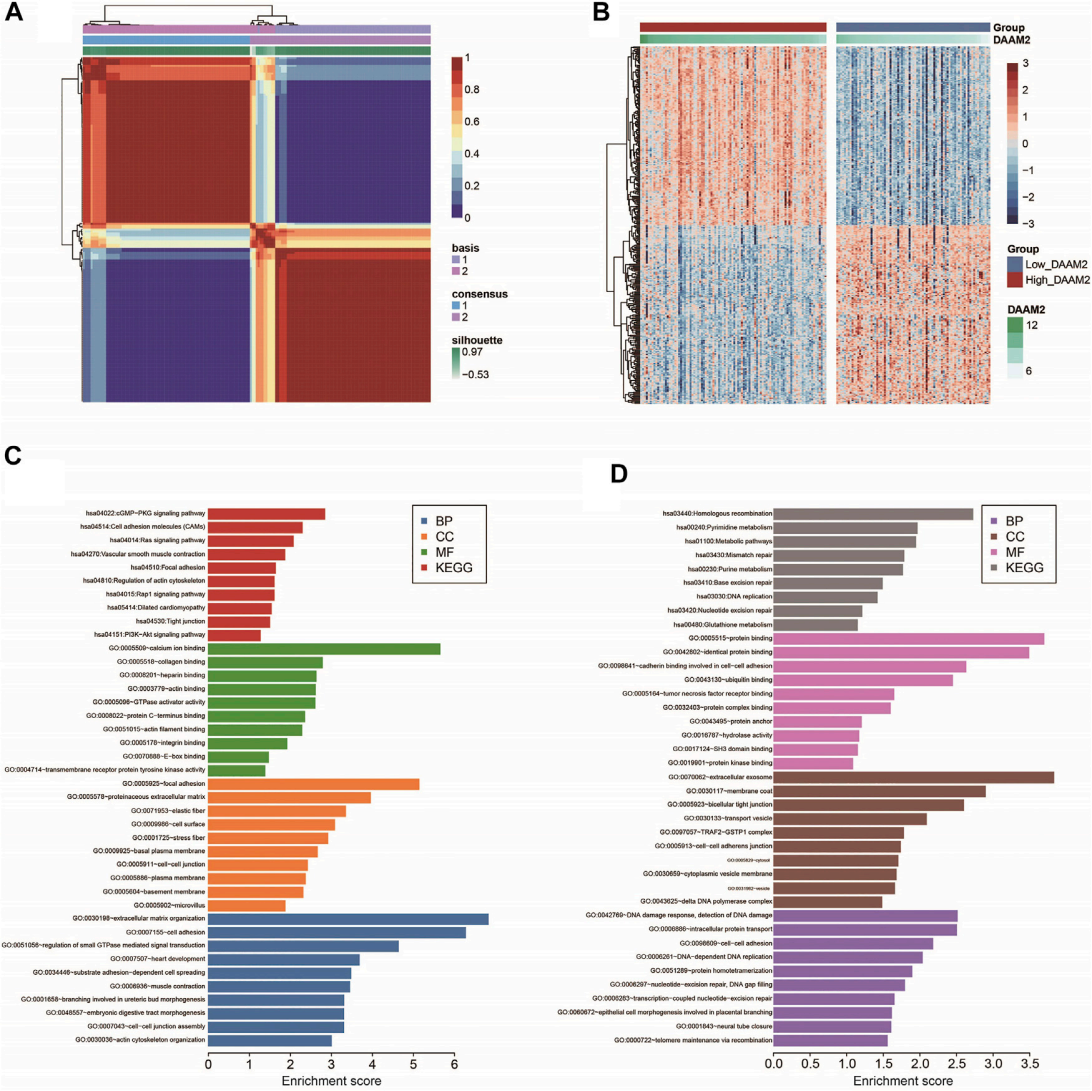
Subgrouping PAAD patients according to DAAM2 expression. **(A)** The consensus map of NMF clustering results in the PAAD cohort. **(B)** Cluster heatmap of positively and negatively correlated genes of DAAM2. **(C)** GO and KEGG analyses of positively correlated genes of DAAM2. **(D)** GO and KEGG analyses of negatively correlated genes of DAAM2.

Next, we used the “bicor” algorithm to evaluate “similarity” between gene expression profiles, and then the nonsmooth NMF algorithm was utilized to classify PAAD patients in each cohort into high- and low-DAAM2 subgroups (Figures 3A,B). These genes that positively or negatively correlated with DAAM2 were submitted to the DAVID database for GO and KEGG analyses. GO enrichment analysis predicted the functional roles of DAAM2 in terms of three aspects: biological process (BP), cellular component (CC) and molecular function (MF). Most positively correlated genes seemed to mediate extracellular matrix organization and were enriched in the cGMP-PKG signaling pathway (Figure 3C). In addition, negatively correlated genes seemed to mediate the DNA damage response and the detection of DNA damage and were enriched in homologous recombination pathways (Figure 3D). To further confirm the role of DAAM2 in mediating cancer immunity in PAAD, we then compared the difference in immunological features of the TME between the High-DAAM2 and Low-DAAM2 subtypes.

### DAAM2 Predicts an Inflamed TME in Pancreatic Adenocarcinoma

**FIGURE 4:**
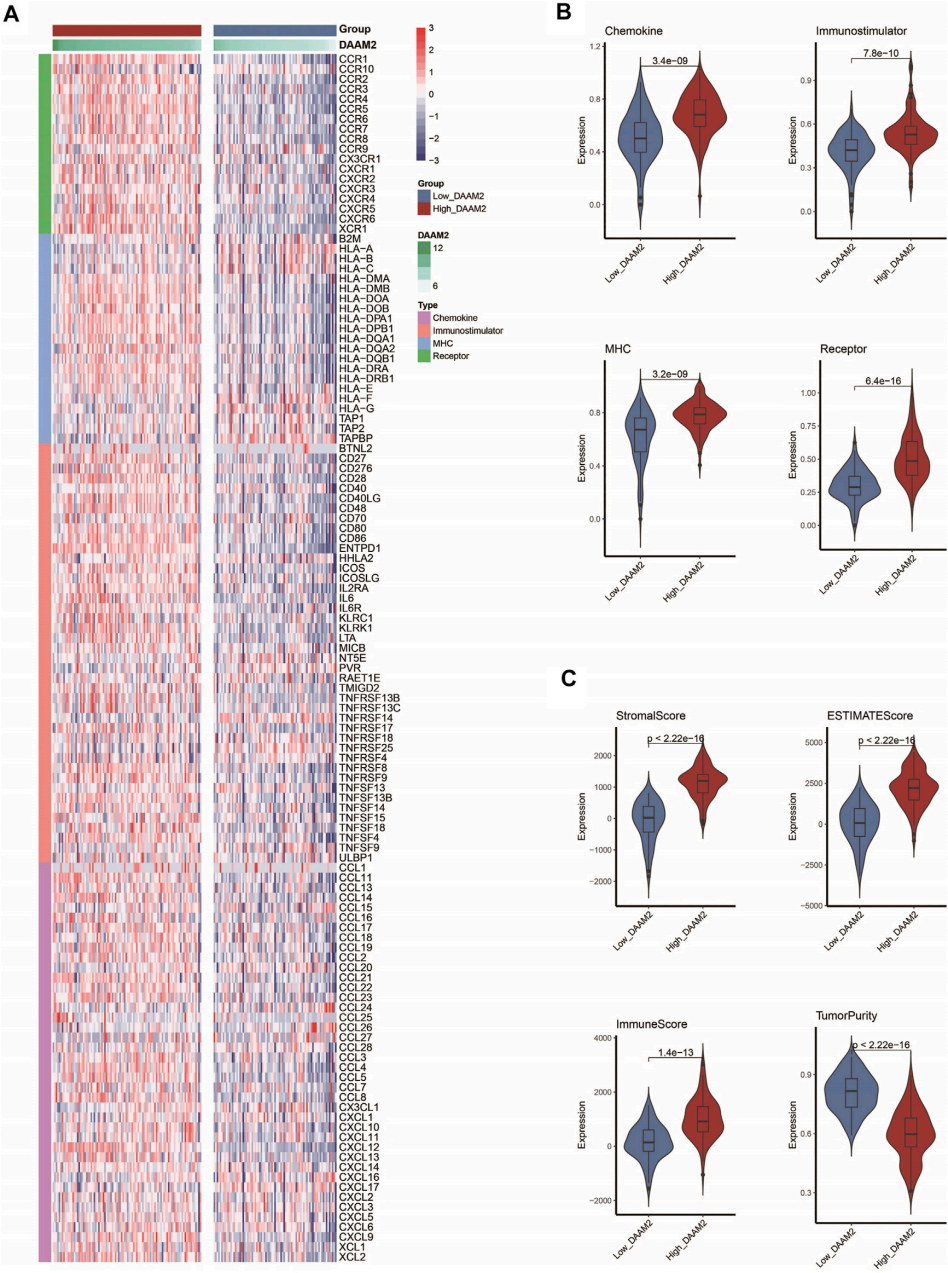
DAAM2 shapes an inflamed TME in PAAD. **(A,B)** Expression levels of 122 immunomodulators in the high and low DAAM2 groups in PAAD. **(C)** Distribution of tumor purity, ESTIMATE score, immune score and stromal score calculated using the ESTIMATE algorithm in the high- and low-DAAM2 groups in PAAD.

We subsequently investigated the immunological role of DAAM2 in PAAD in the TCGA cohort. Many chemokines, paired receptors, MHC molecules and immunomodulators were upregulated in the High-DAAM2 group (Figures 4A,B). Next, the ESTIMATE method was used to assess tumor purity, ESTIMATE score, immune score and stromal score. Compared with the Low-DAAM2 group, the High-DAAM2 group exhibited higher ESTIMATE scores, immune scores and stromal scores but lower tumor purity (Figure 4C). All the results indicated that tumors with high DAAM2 expression were accompanied by increased immune cell infiltration.

**FIGURE 5:**
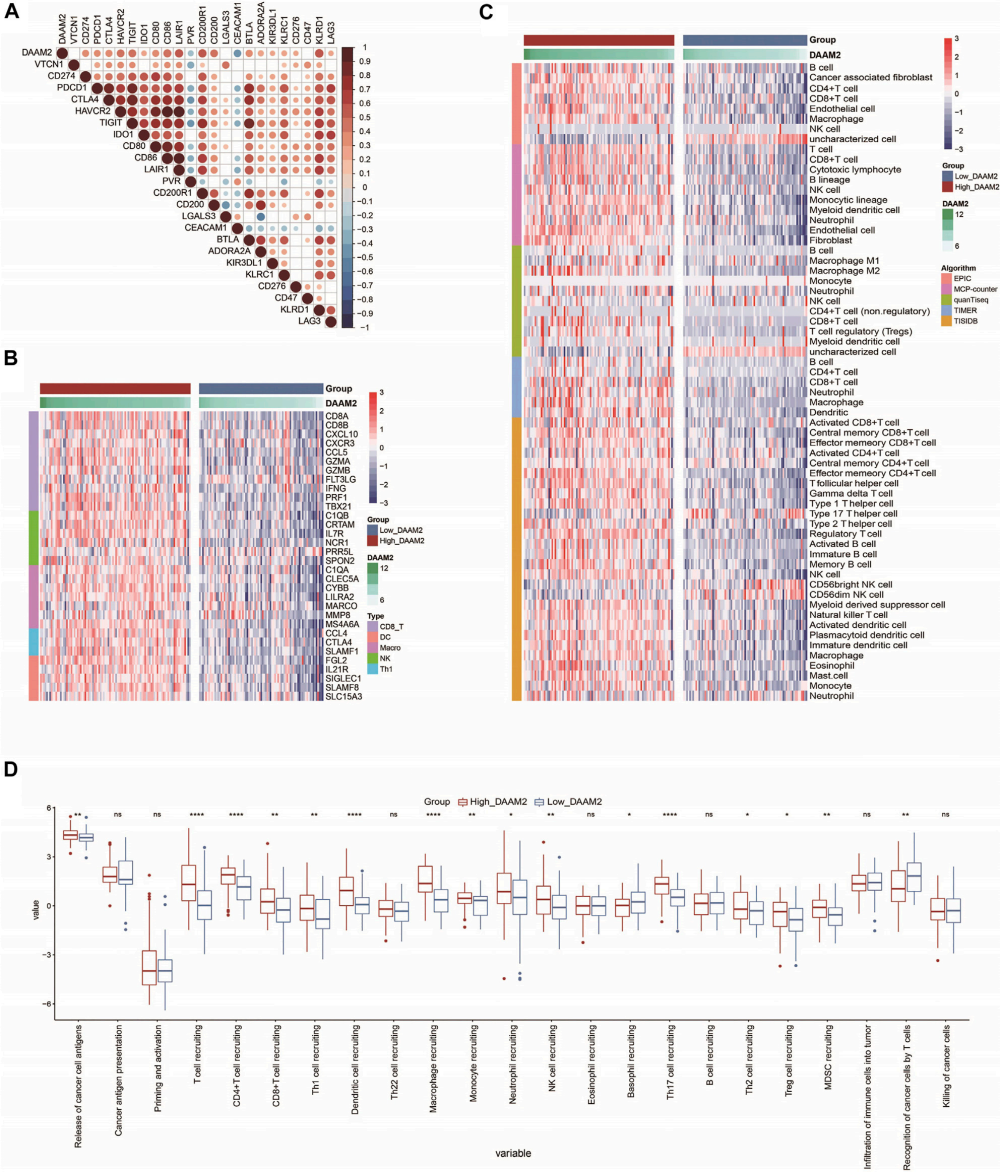
DAAM2 is correlated with increased anticancer immunity in PAAD. **(A)** Correlation between DAAM2 and common inhibitory immune checkpoints. The color and the values indicate the Pearson correlation coefficients. **(B)** Expression levels of the gene markers of the common TIICs in the high and low DAAM2 groups. **(C)** The levels of TIICs calculated using five algorithms in the high- and low-DAAM2 groups. **(D)** The activities of the various steps of the cancer immunity cycle in the high and low DAAM2 groups. Ns: no significant difference; *p < 0.05; **p < 0.01; ***p < 0.001; ****p < 0.0001.

Inhibitory immune checkpoints, such as PD-1/PD-L1, were revealed to be highly expressed in the inflamed TME (Gajewski et al., 2017). DAAM2 was found to be highly correlated with most immune checkpoints in PAAD (Figure 5A). Given the positive correlation between DAAM2 and PD-L1, we next explored whether DAAM2 could regulate PD-L1 expression. The results showed that DAAM2 knockdown significantly inhibited PD-L1 expression (Supplementary Figures S1A–S1C). We next assessed the gene markers of common immune cells and found that these markers were increased in the High-DAAM2 group (Figure 5B). We also estimated the infiltration levels of TIICs based on five independent strategies. The infiltration levels of most immune cells using various algorithms were significantly upregulated in the High-DAAM2 group (Figure 5C). In addition, the activities of the cancer immunity cycle are a direct comprehensive performance of the functions of the chemokine system and other immunomodulators. In the High-DAAM2 group, the activities of the majority of the steps in the cycle were notably upregulated (Figure 5D). In summary, DAAM2 is highly correlated with the inflamed TME in PAAD.

### DAAM2 Predicts Therapeutic Opportunities in Pancreatic Adenocarcinoma

**FIGURE 6:**
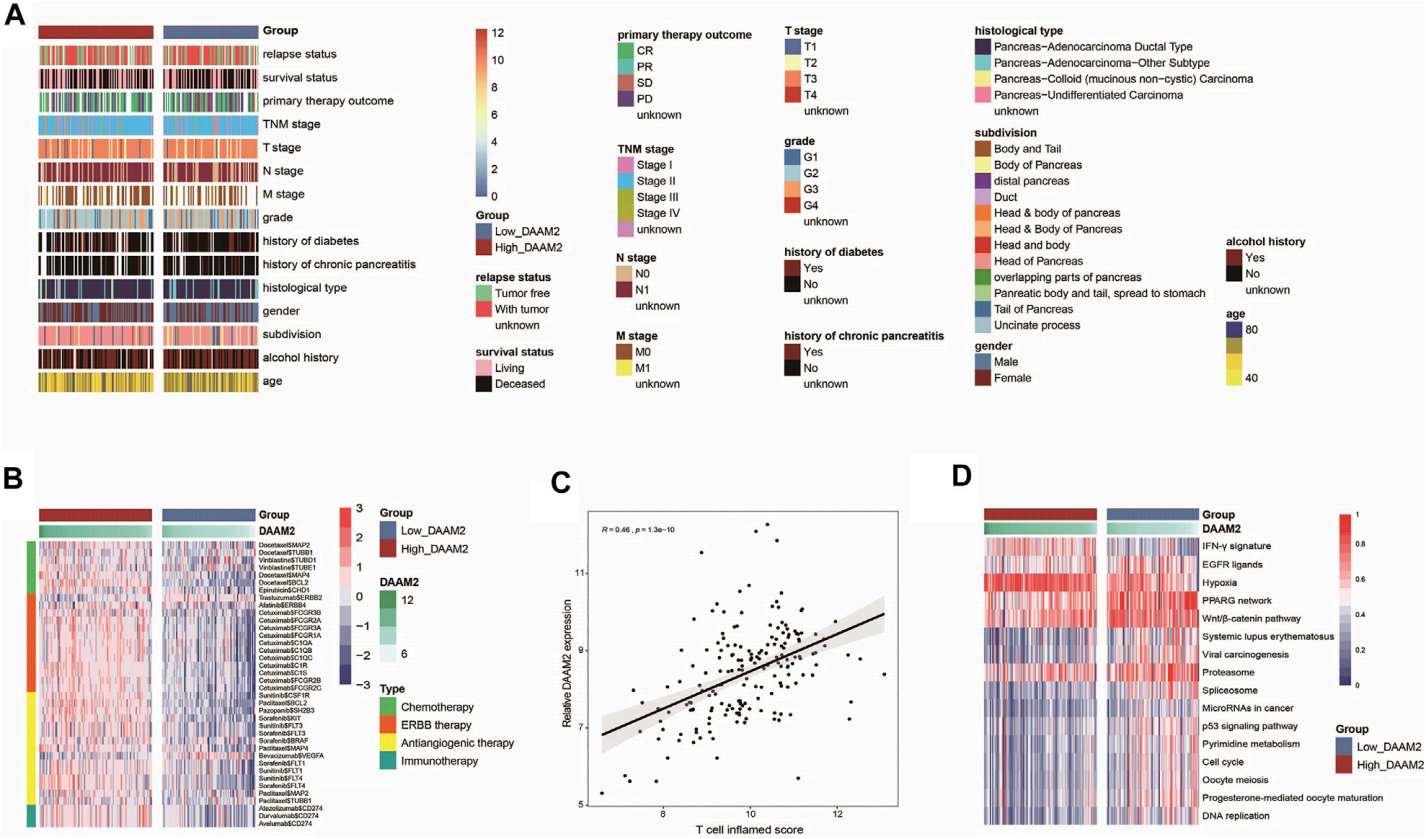
DAAM2 is associated with clinicopathological features in PAAD. **(A)** Correlations between DAAM2 and clinicopathological features in PAAD. **(B)** Correlations between DAAM2 and the drug-target genes extracted from the DrugBank database. **(C)** Correlation between DAAM2 and the T cell inflamed score in PAAD. **(D)** Correlations between DAAM2 and the enrichment scores of several therapeutic signatures related to anticancer immunity.

In principle, patients with high DAAM2 expression may exhibit better responses to various therapies because DAAM2 is associated with an inflamed TME. We first evaluated DAAM2 expression and the clinicopathological features of PAAD. As shown in Figure 5A, DAAM2 was markedly associated with sex but was not related to other features in the TCGA cohort (Figure 6A, Supplementary Table S2). We next evaluated DAAM2 expression and the responses to various therapies. The results from the DrugBank database (https://go.drugbank.com/) indicated significantly higher responses to chemotherapy, anti-EGFR therapy and immunotherapy in the High-DAAM2 group but low responses to anti-ERBB2 and anti-VEGFA therapies (Figure 6B). The T cell inflamed score has been established as an alternative for estimating the clinical response to anti-PD-1 therapy (Ayers et al., 2017). DAAM2 expression was positively related to the T cell inflamed score in the TCGA cohort (Figure 6C). In addition, DAAM2 positively correlated with the enrichment scores of most immunotherapy-positive gene signatures in the TCGA cohort (Figure 6D). Considering the tight association between DAAM2 and hypoxia, we also compared the hypoxia scores in the low and high DAAM2 groups. The results showed that the Buffa, Winter and Ragnum hypoxia scores were higher in the High-DAAM2 group (Supplementary Figures S2A–S2C). Overall, patients with high DAAM2 expression tend to be sensitive to more therapeutic opportunities except for anti-ERBB2 and anti-VEGFA therapies.

### DAAM2 Is Correlated With Immune-Related Microbiota in Pancreatic Adenocarcinoma

**FIGURE 7:**
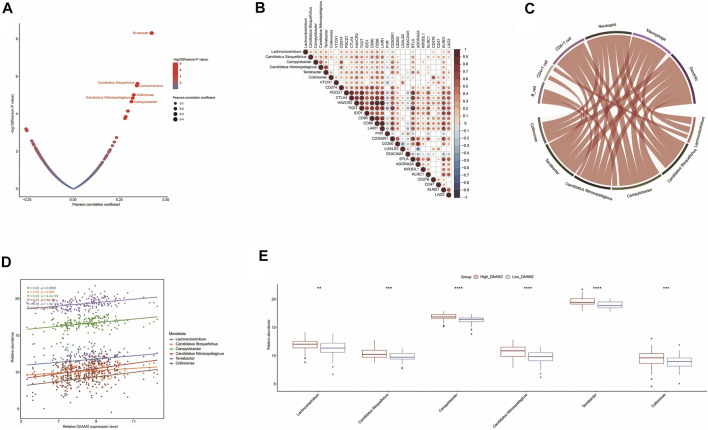
Correlations between DAAM2 and the microbiome signature in PAAD. **(A)** Correlations between the T cell inflamed score and tumor microbial abundances. The dots represent different microbiota. The *Y*-axis represents the Pearson correlation coefficient, while the *X*-axis represents -log_10_(*p* value). **(B)** Correlations between immune-related microbiota abundances and immune checkpoint expression. **(C)** Correlations between immune-related microbiota abundances and TIIC levels estimated by TIMER. **(D)** Correlations between DAAM2 expression and immune-related microbiota abundances. **(E)** Differences in the abundances of immune-related microbiota between the high and low DAAM2 groups.

RNA-seq data could be used to estimate microbial composition in tumor tissues (Dohlman et al., 2021). We systematically analyzed the correlations between microbiota abundance and the T cell inflamed score. A total of six microbiota were extracted with the criterion of Pearson correlation coefficient ≥0.3 or ≤ −0.3: Lachnoclostridium, *Candidatus stoquefichus*, Campylobacter, *Candidatus nitrosopelagicus*, Terrabacter and Collimona, which were all positively correlated with the T cell inflamed score (Figure 7A). Next, the correlations between these immune-related microbiota abundances and the expression of immune checkpoints and immune cell abundance were assessed to validate their correlations with antitumor immunity. As expected, their abundance was positively correlated with the expression of immune checkpoints and immune cell abundance (Figures 7B,C). These results implied that high abundances of these six microbiotas were correlated with a higher response to immunotherapy. Encouragingly, the abundances of these six microbiotas were positively correlated with DAAM2 expression and were higher in the High-DAAM2 group (Figure 7D). Taken together, these results suggest that DAAM2 expression is correlated with the response to immunotherapy from the angle of correlations with immune-related microbiota.

## Discussion

There are two DAAM proteins, but to date, the majority of studies have focused on DAAM1. In addition to breast cancer (Zhu et al., 2012; Mei et al., 2019; Mei et al., 2020b; Mei et al., 2021), DAAM1 also plays oncogenic roles in multiple tumors. For example, DAAM1 is overexpressed in gastric cancer and promotes tumor progression by regulating the ERK and AKT signaling pathways (Zhang et al., 2021). In addition, Src-mediated activation of DAAM1 induced actin polymerization and thus promoted lung cancer metastasis (Li et al., 2019). Similar to DAAM1, DAAM2 can transduce dishevelled-dependent Wnt/PCP signals to the RHOA signaling cascade (Katoh, 2005). In addition to participating in noncanonical Wnt signaling, DAAM2 can stabilize dishevelled 3/Axin2 binding, leading to the enhanced intracellular assembly of dishevelled 3/Axin2 complexes, thus mediating the functions of canonical Wnt signaling (Lee and Deneen, 2012). The role of DAAM2 in cancers has been preliminarily summarized. As previously described, DAAM2 accelerated the progression of glioma and hepatocellular carcinoma by facilitating the degradation of VHL (Zhu et al., 2017; Fang et al., 2020). In addition, DAAM2 is essential for the formation of actin-rich filopodia structures and then contributes to the increased capacity of migration and invasion (Lynch et al., 2013). However, the expression of DAAM2 and its immunological correlation have not yet been explored.

In our research, we first analyzed the immunological correlation of DAAM2 across cancers utilizing large-scale RNA-seq data and then validated the expression of DAAM2 in PAAD. The results suggested a potential role of DAAM2 as an immune-related indicator in human cancers, especially PAAD. The results also revealed that DAAM2 was upregulated in PAAD tissues. The oncogenic role of DAAM2 has been confirmed in glioma and hepatocellular carcinoma (Zhu et al., 2017; Fang et al., 2020), but the biological function of DAAM2 in PAAD needs to be further explored.

Formin proteins are well known as regulators of microfilament assembly and cell migration (Le et al., 2020). However, whether these proteins are related to antitumor immunity and the TME has not been considered. Previous research revealed that FMNL proteins, a subfamily of Formin proteins, were correlated with immune cell infiltration in gastric cancer (Nie et al., 2020). In addition, in an immune-related classification strategy for cervical cancer, DAAM2 was used as one of the critical genes for risk demarcation (Mei et al., 2020a). Moreover, several studies uncovered the direct connection between DAAM2 and hypoxia, a powerful regulatory factor for immunity. On the one hand, DAAM2 facilitated the degradation of VHL and upregulated HIF-1α expression (Zhu et al., 2017; Fang et al., 2020); on the other hand, DAAM2 was upregulated by hypoxia in the circulation and placenta (de Alwis et al., 2021). These findings suggest that Formin proteins, especially DAAM2, are associated with antitumor immunity in cancer.

As an important finding, the expression of DAAM2 was correlated with almost all immunomodulator and TIIC levels in PAAD. Specifically, we found that DAAM2 was positively correlated with the expression of critical immunomodulators, such as CCL5, CXCL9, and CXCL10, as well as the activities of the cancer-immunity cycle. In addition, the immunological role of DAAM2 was opposite to several reported immunosuppressive oncogenic pathways, such as the *β*-catenin and PPAR-*γ* pathways (Spranger et al., 2015; Korpal et al., 2017). These pathways have been revealed to suppress the infiltration of TIICs by decreasing the expression of immunomodulators, shaping a noninflamed TME. DAAM2 was markedly negatively correlated with the enrichment scores of these oncogenic pathways but positively correlated with immunopromotive pathways, such as the IFN-γ signature, EGFR ligands and hypoxia. In addition, DAAM2 was correlated with emerging immunobiomarkers in PAAD, namely, immune-related microbiota. However, it remains to be further clarified whether DAAM2 is merely a biomarker for tumor immunogenicity or has potential regulatory effects on antitumor immunity in PAAD.

## Conclusion

This study reveals that DAAM2 is upregulated and shapes an inflamed TME in PAAD, which can also predict immune and clinical phenotypes. Additionally, DAAM2 is correlated with immune-related microbiota in PAAD. Overall, DAAM2 might be a potential biomarker for assessing tumor immunogenicity and guiding immunotherapy.

## Data Availability

The original contributions presented in the study are included in the article/Supplementary Material, further inquiries can be directed to the corresponding authors.

## Abbreviations

PAAD, pancreatic adenocarcinoma; VHL, Von Hippel-Landau; HIF-1α, hypoxia-inducible factor 1α; RNA-seq, RNA sequencing; NMF, nonnegative matrix factorization; GO, Gene Ontology; KEGG, Kyoto Encyclopedia of Genes and Genomes; TIICs, tumor-infiltrating immune cells; ssGSEA, single sample gene set enrichment analysis; TMA, tissue microarray; IHC, immunohistochemistry; IRS, immunoreactivity score; BP, biological process; CC, cellular component; MF, molecular function.
