# Supplementary material for: Dishevelled-Associated Activator of Morphogenesis 2 (DAAM2) Predicts the Immuno-Hot Phenotype in Pancreatic Adenocarcinoma
Source: Front Mol Biosci. 2022 Feb 24;9:750083. doi: 10.3389/fmolb.2022.750083 (PMC8907973; doi:10.3389/fmolb.2022.750083)
Supplement: Supplementary file 2 [file Table2.DOCX]

Table S2. Correlations between DAAM2 and clinic-pathological features in PAAD.

| Clinic-pathological features | P value |
| --- | --- |
| Relapse status | 1.000 |
| Survival status | 0.670 |
| Primary therapy outcome | 0.293 |
| TNM stage | 0.862 |
| N stage | 0.106 |
| M stage | 1.000 |
| T stage | 0.816 |
| Grade | 0.617 |
| History of diabetes | 0.372 |
| History of chronic pancreatitis | 0.525 |
| Histological type | 0.218 |
| Subdivision | 0.142 |
| Gender | 0.009 |
| Alcohol history | 0.177 |
| Age | 0.118 |
