# Supplementary material for: Dishevelled-Associated Activator of Morphogenesis 2 (DAAM2) Predicts the Immuno-Hot Phenotype in Pancreatic Adenocarcinoma
Source: Front Mol Biosci. 2022 Feb 24;9:750083. doi: 10.3389/fmolb.2022.750083 (PMC8907973; doi:10.3389/fmolb.2022.750083)
Supplement: Supplementary file 3 [file Table3.DOCX]

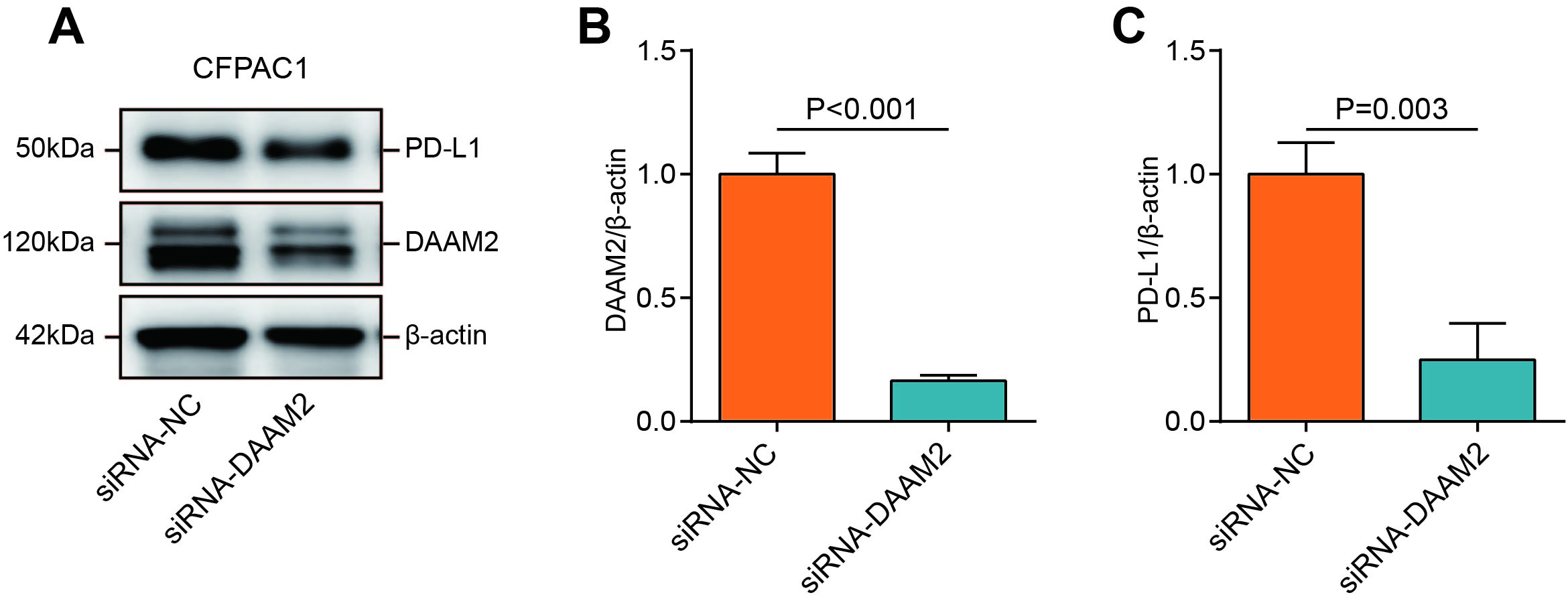


Figure S1. Effects of DAAM2 on PD-L1 expression in CFPAC-1 cells.

(A) The protein levels of DAAM2 and PD-L1 in CFPAC-1 cells with DAAM2 knockdown examined by Western blotting. (B, C) Quantitative gray analysis of DAAM2 and PD-L1.
