# Supplementary material for: Dishevelled-Associated Activator of Morphogenesis 2 (DAAM2) Predicts the Immuno-Hot Phenotype in Pancreatic Adenocarcinoma
Source: Front Mol Biosci. 2022 Feb 24;9:750083. doi: 10.3389/fmolb.2022.750083 (PMC8907973; doi:10.3389/fmolb.2022.750083)
Supplement: Supplementary file 4 [file Table4.DOCX]

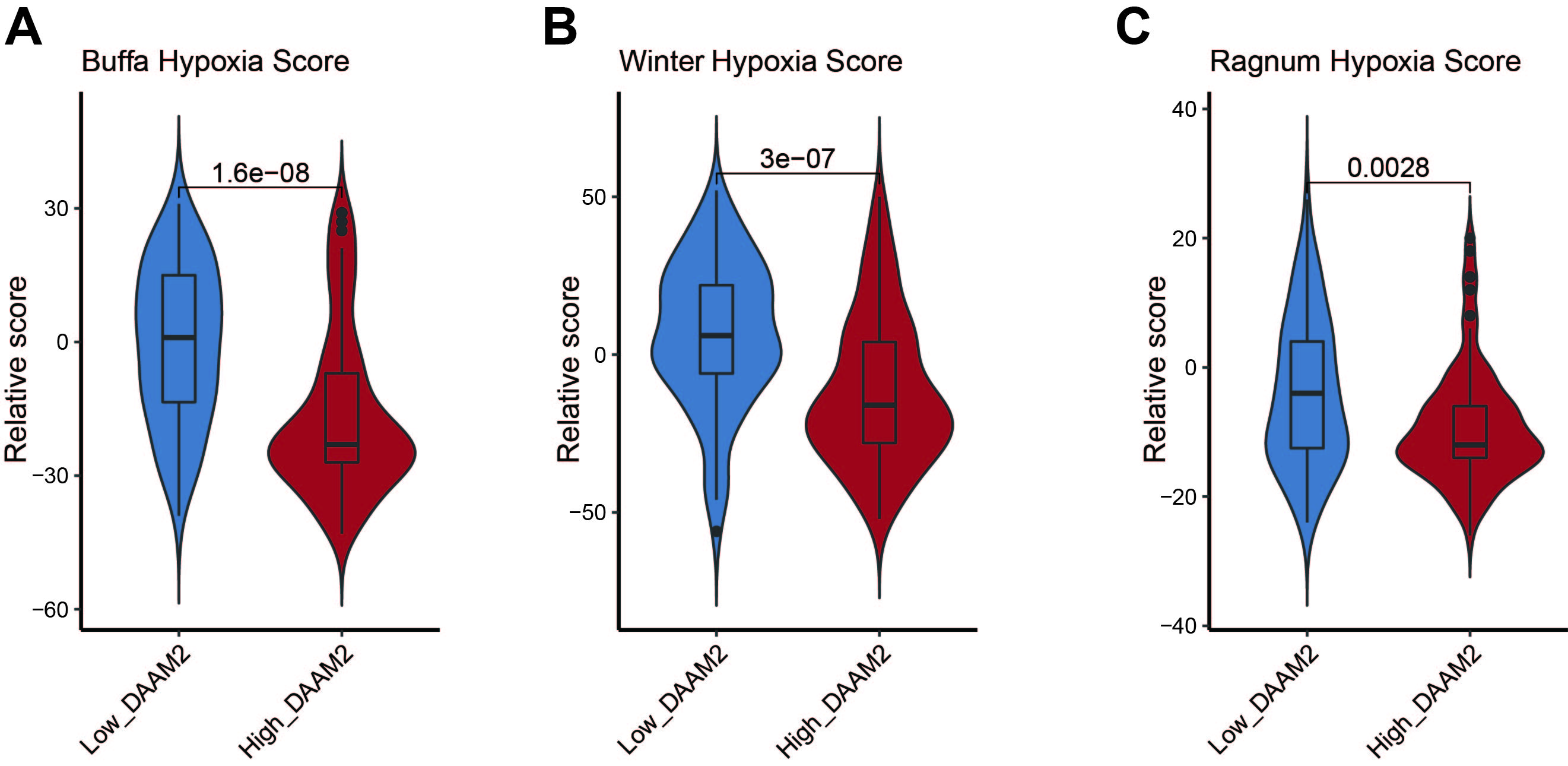


Figure S2. Differences in (A) Buffa, (B) Winter, (C) Ragnum Hypoxia Score between the high and low DAAM2 groups.
